# Total, Fresh, Lean, and Fresh Lean Beef Consumption in Relation to Nutrient Intakes and Diet Quality among U.S. Adults, 2005–2016

**DOI:** 10.3390/nu11030563

**Published:** 2019-03-06

**Authors:** Ruopeng An, Sharon Nickols-Richardson, Reginald Alston, Sa Shen, Caitlin Clarke

**Affiliations:** 1 Department of Kinesiology and Community Health, University of Illinois, Urbana-Champaign, IL 61820, USA; alston@illinois.edu (R.A.); vitosky@illinois.edu (C.C.); 2 Brown School, Washington University in St. Louis, MO 63130, USA; 3 Department of Food Science and Human Nutrition, Division of Nutritional Sciences, University of Illinois Extension, University of Illinois at Urbana-Champaign, IL 61820, USA; nickrich@illinois.edu; 4 College of Applied Health Sciences, University of Illinois, Urbana-Champaign, IL 61820, USA; sashen2@illinois.edu

**Keywords:** beef consumption, nutrient intakes, diet quality, fresh lean beef, red meat consumption, nutrition guidelines

## Abstract

(1) Background: This study assessed the influence of beef consumption on nutrient intakes and diet quality among U.S. adults. (2) Methods: Nationally-representative sample (*n* = 27,117) from 2005–2016 National Health and Nutrition Examination Survey was analyzed. First-difference estimator addressed confounding bias from time-invariant unobservables (e.g., eating habits, taste preferences) by using within-individual variations in beef consumption between 2 nonconsecutive 24 h dietary recalls. (3) Results: Approximately 54%, 39%, 12%, and 7% of U.S. adults consumed beef, lean beef, fresh beef, and fresh lean beef, respectively. Overall diet quality measured by the Health Eating Index-2015 (HEI-2015) score among beef, fresh beef, lean beef, and fresh lean beef consumers was lower than beef non-consumers. Regression analyses found that beef, fresh beef, lean beef, and fresh lean beef consumption was associated with higher daily intakes of total energy, protein, sodium, choline, iron, selenium, zinc, phosphorus, and multiple B vitamins. Beef, fresh beef, and lean beef consumption but not fresh lean beef consumption was associated with higher saturated fat intake. Beef consumption was not found to be associated with overall dietary quality measured by the HEI-2015 score. (4) Conclusions: Beef consumers may increase the intake of fresh and lean beef over total beef consumption to maximize the nutritional gains from beef portions while minimizing the resulting increases in energy, saturated fat, and sodium.

## 1. Introduction

Beef is a staple of the U.S. diet. In 2016, U.S. adults averaged 56 pounds of beef consumption [1]. Dietary animal protein is a primary source of high biological value protein, iron, zinc, multiple B vitamins and other essential nutrients [2]. As a primary source of dietary animal protein, beef consumption, especially fresh and lean beef, holds the potential to improve diet quality among U.S. adults. On the other hand, beef contains saturated fat and may be prepared or cooked in ways (e.g., certain processed beef products) that increase the presence of unhealthful substances [3]. Evidence from some prospective cohort studies and randomized controlled trials suggests that eating patterns that include lower intake of red meats as well as processed meats are associated with a reduced risk of obesity, type 2 diabetes, cardiovascular disease, and some types of cancer in adults [4].

Previous research suggests that U.S. adults who consume moderate amounts of beef have similar diet quality to non-beef consumers [5]. Importantly, groups who specifically chose to consume beef with the highest lean meat and lowest fat content had higher intakes of protein as well as vitamins B_3_, B_6_, B_12_, iron, phosphorus, and zinc [5]. The same group also had low intakes of total fat, carbohydrates, and total energy. Two other studies, which focused on children and adolescents, supported these findings concerning significant contribution of lean beef consumption to vitamin and protein intakes for children and adolescents [6,7]. An earlier examination of the U.S. National Health and Nutrition Examination Survey (NHANES) 1999–2004 waves found that half of U.S. adults consumed beef during the dietary recall [8]. Adult participants 19–50 years acquired a small to moderate amount of protein, saturated fat, vitamin B_12_, zinc, and iron from lean beef consumption [8]. Another study, which also corroborated these findings, noted that the highest lean and lowest fat beef consumers had higher intakes of total vegetables as well as dark green/orange vegetables, legumes, lower milk, and lower solid fat [9]. These findings have been reproduced in different racial/ethnic sub-populations including African-American, Hispanic, Asian, and Caucasian—particularly in relation to the contribution of beef to zinc, vitamin B_12_, and iron intakes [10]. Nevertheless, these data points do not necessarily lead to the conclusion that in general people should increase their beef consumption.

Previous research links red meat consumption, especially processed meat consumption, to increased risk of type 2 diabetes, stroke, and premature mortality [11,12]. Moreover, current research on beef consumption is concerned with the carbon footprint of producing and consuming beef, leading to recommendations for a general reduction in beef consumption [13,14]. Others argue that the contribution of red meat to diet quality is under-appreciated—especially in relation to protein and micronutrients less commonly found in plant-based alternatives [15]. In addition, several studies suggest that recommendations to reducing beef consumption are too strict because of a lack of distinction between processed and unprocessed red meat [16]. Furthermore, recommendations to restrict beef intake have been largely based on concerns over the relationship between saturated fatty acids and cardiovascular health, which more recent literature suggests is a significantly more complex issue than previously understood [17,18]. Instead, some of the previous literature argues for a re-evaluation of nutrition and dietary recommendations, based on unprocessed and lean meat, arguing that lean beef can be part of a healthy diet [15,16,18].

Building on previous literature, the purpose of this study is to assess beef consumption in relation to nutrient intakes and diet quality among U.S. adults aged 18 years and older. It contributes to the literature in three aspects. First, to our knowledge, it serves as the first study that differentiates between fresh beef, lean beef, and fresh lean beef, and examines their distinct relationship with daily nutrient intake and diet quality. Second, it produces population-level estimates by examining a large, nationally-representative nutrition survey, with a time span of 12 years from 2005 to 2016. Third, it adopts a first-difference modeling approach that eliminates the potential confounding bias from the differences in time-invariant individual characteristics. The study hypothesized that beef consumption, in particular, fresh and lean beef consumption, would be positively associated with higher daily intakes of protein, choline, iron, selenium, zinc, phosphorus, and vitamins B_2_, B_3_, B_6_, and B_12_. These 10 nutrients are particularly rich in beef products [8,19]. Beef consumption was also hypothesized to be associated with higher daily intakes of total energy, saturated fat, and sodium, but would be more modest for fresh and lean beef consumption.

## 2. Materials and Methods

### 2.1. Survey Setting and Participants

The U.S. NHANES is a program of studies conducted by the National Center for Health Statistics (NCHS) to assess the health and nutritional status of children and adults. The program began in the early 1960s and periodically conducted separate surveys focusing on different population groups or health topics. Since 1999, the NHANES has been conducted continuously in 2-year cycles, and has a changing focus on a variety of health and nutrition measurements. A multi-stage probability sampling design has been used to select participants who are representative of the civilian, non-institutionalized U.S. population. Certain population sub-groups are oversampled to increase the reliability and precision of health status indicator estimates for these groups. Detailed information regarding the NHANES sampling design, questionnaires, clinical measures, and individual-level data can be found elsewhere [20].

### 2.2. Dietary Interview

Except for the NHANES 1999–2000 wave where all participants were asked to complete a single 24 h dietary recall, all subsequent waves incorporated two dietary recalls, with the first collected in-person, and the second by telephone 3 to 10 days later. In both interviews, each food or beverage item, and corresponding quantity consumed by a participant from midnight to midnight on the day before the interview, were recorded. The in-person dietary recall (day 1) was conducted by trained dietary interviewers in the mobile examination center (MEC) with a standard set of measuring guides. These tools aimed to help the participant accurately report the volume and dimensions of the food/beverage items consumed. Upon completion of the in-person interview, participants were provided measuring cups, spoons, a ruler and a food model booklet, which contained 2-dimensional drawings of the various measuring guides available in the MEC, to use for reporting dietary intake during the telephone interview (day 2). Following the dietary interview, the caloric and nutrient contents of each reported food and/or beverage item were systematically coded with the U.S. Department of Agriculture (USDA) Food and Nutrient Database for Dietary Studies (FNDDS). Access restrictions apply to the day 2 dietary recall data collected in the NHANES 2001–2002 wave, whereas dietary data for both recall days are released to the public for all subsequent waves.

### 2.3. Beef Consumption

Each food item consumed is assigned an 8-digit FNDDS code in the NHANES. Beef products occupy the codes 21000000–21800000. However, FNDDS codes do not differentiate between fresh and/or lean beef. We therefore linked FNDDS codes to the USDA National Nutrient Database for Standard Reference (SR), which assigns a 5-digit Nutrient Databank (NDB) number to each food item. The NDB numbers are linked to the FNDDS codes in the FNDDS link files. Beef is a unique food group classified in the SR, and each beef product is associated with a detailed text description. Fresh beef refers to beef products that do not contain any artificial flavor or flavoring, coloring ingredient, chemical preservative, or any other artificial or synthetic ingredient; and the products and their ingredients are not more than minimally processed (e.g., ground). We identified fresh beef products using the keywords “fresh” or “raw”, and lean beef products using “lean” in the description. Fresh lean beef products are beef products that are both fresh and lean. The SR defines fresh lean beef as fresh beef, containing less than 10 g of fat, 4.5 g of saturated fat, and 95 mg of cholesterol per 100 g of product. To estimate the ounce-equivalents of beef consumption, we further merged the NHANES data with the corresponding Food Patterns Equivalents Database (FPED). A new version of the FPED can be developed for each NHANES wave. FPED converts the foods and beverages in the FNDDS to the USDA food patterns (FPs) components, and the FPs are measured as ounce-equivalents for protein foods. Due to the modifications of the FPs classifications in FPED over the years, we adopted the most recent version of FPs classifications that has been consistent since the NHANES 2005—2006 wave.

A beef consumer is defined as an adult NHANES participant who consumed any beef products on either dietary recall day. Analogously, a fresh, lean, or fresh lean beef consumer is defined as an adult participant who consumed any fresh, lean, or fresh lean beef products on either dietary recall day, respectively. These three consumer groups are not mutually exclusive and an individual may simultaneously qualify for one, two, or all three categories. In contrast, a beef non-consumer is defined as an adult participant who consumed no beef products on both dietary recall days.

### 2.4. Diet Quality

The Healthy Eating Index (HEI)-2015 was developed by the USDA as a measure of dietary quality in accordance with the Dietary Guidelines for Americans (DGA), 2015–2020 [21,22]. It consists of 13 components: Total fruit, whole fruit, total vegetables, greens and beans, whole grains, dairy, total protein foods, seafood and plant proteins, fatty acids, refined grains, sodium, added sugars, and saturated fats. With a maximum score of 100, a higher HEI-2015 score reflects closer adherence to the DGA. We calculated each NHANES participant’s HEI-2015 score on either dietary recall day using the FPED and following the procedures established by the USDA and the National Cancer Institute [22].

### 2.5. Nutrient Intakes

In the dietary recall data, energy derived from each consumed food/beverage item was recorded based on the quantity of food/beverage reported and the corresponding energy contents. We calculated the daily caloric intake (kcal) and daily intakes of protein (g), saturated fat (g), sodium (mg), choline (mg), iron (mg), selenium (µg), zinc (mg), phosphorus (mg), vitamin B_2_ (mg), vitamin B_3_ (mg), vitamin B_6_ (mg), and vitamin B_12_ (µg) from beef products alone as well as from all foods/beverages on either dietary recall day among beef consumers, fresh beef consumers, lean beef consumers, fresh lean beef consumers, and beef non-consumers.

### 2.6. Individual Characteristics

The following individual characteristics were reported for U.S. adults aged 18 years and older: sex, age (stratified into two age groups: 18–64 years of age and 65 years of age and older), race/ethnicity (non-Hispanic white, non-Hispanic African American, non-Hispanic other race or multi-race, and Hispanic), education (high school and below, and college and above), marital status (married, divorced/separated/widowed, and never married), household income (income to poverty ratio (IPR) < 130%, 130% ≤ IPR < 300%, and IPR ≥ 300%), smoking status (non-smoker, and former or current smoker), self-rated health (good or excellent health, and fair or poor health), chronic conditions (diabetes, arthritis, coronary artery disease, stroke, and cancer), survey wave, and obesity status. Participants’ body height and weight were measured by stadiometer and digital scale in the MEC. Body mass index (BMI) is defined by weight in kilograms divided by height in meters squared (kg/m^2^). Adult obesity was defined as BMI ≥ 30 kg/m^2^ based on the international classification of adult BMI values [23].

### 2.7. Sample Size

This study used individual-level data from the NHANES 2005–2006, 2007–2008, 2009–2010, 2011–2012, 2013–2014, and 2015–2016 waves. Among a total of 28,704 U.S. adults aged 18 years and older who participated in the 24 h dietary recalls, 1587 that were pregnant, lactating, and/or on a special diet to lose weight at the time of interview were excluded, resulting in a final sample of 27,117 participants.

### 2.8. Statistical Analyses

Using descriptive statistics, we summarized individual characteristics of beef consumers, fresh beef consumers, lean beef consumers, fresh lean beef consumers, and beef non-consumers and their daily caloric intake and daily intakes of protein, saturated fat, sodium, choline, iron, selenium, zinc, phosphorus, vitamin B_2_, vitamin B_3_, vitamin B_6_, and vitamin B_12_ from beef products alone as well as from all foods/beverages.

Logistic regressions were performed to estimate the adjusted odds ratios of beef, fresh beef, lean beef, or fresh lean beef consumption with respect to individual characteristics among NHANES adult participants. The dependent variables were dichotomous variables for any beef, fresh beef, lean beef, or fresh lean beef consumption on either dietary recall day.

A first-difference estimator was performed on beef, fresh beef, lean beef, and fresh lean beef consumers using data from their day 1 and day 2 dietary interviews, that provided two observations per person. The outcome (e.g., daily total caloric intake and zinc intake) of participant *i* on day *t* (t=1, 2) is denoted by $y_{it}$. We let vector $X_{i}$ represent the set of variables that vary by participant, but remain constant within-participant between the two dietary interviews (e.g., sex and race/ethnicity). Given the short recall time interval of 3–10 days, $X_{i}$ includes individual characteristics that vary only in the longer term, such as age, education attainment, income level, and body weight. Continuous variable $beef_{it}$ denotes daily beef (or fresh, lean, or fresh lean beef) consumption in the unit of ounce-equivalents by participant i on day *t*. Indicator variable $we_{it}$ denotes whether day *t* was a weekend (Friday, Saturday or Sunday).

A pooled cross-sectional setup (a conventional regression that treats repeated measures within each study subject as independent observations) specifies the outcome $y_{it}$ as a function of an unobservable term that varies by participant $\alpha_{i}$, observable variables that vary by participant $X_{i}$, observable variables that vary within-participant between the two dietary interviews $beef_{it}$ and $we_{it}$, and an independently-distributed unobservable disturbance term $\varepsilon_{it}$.

(1) $$y_{it}=\mu X_{i}+\beta_{1}beef_{it}+\beta_{2}we_{it}+\alpha_{i}+\varepsilon_{it}$$

Due to the presence of the unobservable term $\alpha_{i}$ (e.g., eating habits, taste preferences), estimating Equation (1) by controlling for the observables $X_{i}$ only is prone to omitted variable bias. The first-difference estimator eliminates the bias by taking the difference between the 2 days of data within each participant, so that $\alpha_{i}$ and $\mu X_{i}$ that are common within-participant are removed.

(2) $$y_{i1}-y_{i2}=\beta_{1}\left(beef_{i1}-beef_{i2}\right)+\beta_{2}\left(we_{i1}-we_{i2}\right)+\left(\varepsilon_{i1}-\varepsilon_{i2}\right)$$

Equation (2) was estimated for each outcome variable (i.e., HEI-2015 score, daily total caloric intake, and daily intakes of protein, saturated fat, sodium, choline, iron, selenium, zinc, phosphorus, vitamin B_2_, vitamin B_3_, vitamin B_6_, and vitamin B_12_) and each type of beef consumption (i.e., beef, fresh beef, lean beef, and fresh lean beef). There are 14 outcome variables and 4 types of beef consumption so that a total of 56 regressions were estimated.

The NHANES 2005–2016 multi-wave complex survey design was accounted for in both descriptive statistics and regression analyses. The false discovery rate (FDR) was used to adjust for multiple comparisons in regression estimates. All statistical procedures were performed in Stata 15.1 SE version (StataCorp, College Station, TX). A *p*-value < 0.05 was considered as being statistically significant.

### 2.9. Ethnical Approval

The NHANES was approved by the NCHS Research Ethics Review Board. This study used the NHANES de-identified public data and was exempt from human subjects review by the University of Illinois at Urbana-Champaign Institutional Review Board.

## 3. Results

Table 1 reports individual characteristics of 2005–2016 NHANES adult beef consumers and non-consumers. Beef, fresh beef, lean beef, and fresh lean beef consumers occupied 53.6%, 11.6%, 39.3%, and 6.7% of the study sample, respectively. They averaged daily consumption of 2.1 ounce-equivalents of beef, 1.8 ounce-equivalents of fresh beef, 2.2 ounce-equivalents of lean beef, and 2.0 ounce-equivalents of fresh lean beef. Daily total energy intake averaged 300.7, 217.9, 166.9, and 159.6 kcal from beef, fresh beef, lean beef, and fresh lean beef products, respectively. Beef consumers daily acquired 22.9 g of protein, 5.7 g of saturated fat, 664.6 mg of sodium, 74.8 mg of choline, 2.8 mg of iron, 25.7 µg of selenium, 4.4 mg of zinc, 232.4 mg of phosphorus, 0.25 mg of vitamin B_2_, 5.30 mg of vitamin B_3_, 0.36 mg of Vitamin B_6_, and 1.74 µg of vitamin B_12_ from beef products. Daily intakes of saturated fat and sodium from fresh beef (2.8 g of saturated fat and 310.9 mg of sodium), lean beef (3.9 g of saturated fat and 419.8 mg of sodium), and fresh lean beef products (2.3 g of saturated fat and 341.5 mg of sodium) were noticeably lower than those acquired from beef products. Other nutrient intakes from fresh beef, lean beef, and fresh lean beef products were also modestly lower than those acquired from beef products.

Diet quality among beef, fresh beef, lean beef, and fresh lean beef consumers was lower than beef non-consumers, whereas their daily intakes of total energy, protein, saturated fat, sodium, and all the other nutrients were higher than non-consumers. Beef, fresh beef, lean beef, and fresh lean beef consumers averaged an HEI-2015 score of 49.8, 50.0, 50.0, and 50.5, respectively, compared to 53.3 among beef non-consumers. Beef consumers averaged 2209.9 kcal of daily total energy, 88.0 g of protein, 28.4 g of saturated fat, and 3679.2 mg of sodium, compared to 1977.7 kcal of daily total energy, 76.4 g of protein, 23.8 g of saturated fat, and 3290.2 mg of sodium among beef non-consumers. The variable means pertaining to daily total energy/nutrient intakes and diet quality, between beef consumers and non-consumers are all statistically significant at *p*-value < 0.001.

Table 2 reports the adjusted odds ratios of beef, fresh beef, lean beef, and fresh lean beef consumption estimated from logistical regressions. Females were less likely to consume beef, fresh beef, and lean beef products than males. Older adults aged 65 years and older were less likely to consume beef but more likely to consume fresh beef and fresh lean beef compared to their younger counterparts. African Americans were less likely to consume beef and lean beef than whites, whereas Hispanic were more likely to consume beef and lean beef but less likely to consume fresh beef than whites. People with college educations and above were less likely consume beef, fresh beef, lean beef, and fresh lean beef than those with high school or lower education. Compared to their married counterparts, those divorced/separated/widowed or never married were less likely to consume beef, lean beef, and fresh lean beef (except for those divorced/separated/widowed). People with obesity were more likely to consume beef, fresh beef, lean beef, and fresh lean beef than their non-obese counterparts. Income, smoking, self-rated health, and chronic diseases were largely unassociated with beef consumption.

Table 3 reports the estimated effects (i.e., associations) of beef, fresh beef, lean beef, and fresh lean beef consumption on daily energy/nutrient intake and diet quality using the first-difference estimator. An increase in beef, fresh beef, lean beef, and fresh lean beef consumption was found to be associated with an increase in intakes of total energy, protein, sodium, choline, iron, selenium, zinc, phosphorus, vitamin B_2_, vitamin B_3_, and vitamin B_6_. An increase in beef, fresh beef, and lean beef consumption was found to be associated with an increase in daily intakes of saturated fat and vitamin B_12_. In contrast, no association linking fresh lean beef consumption with daily intakes of saturated fat and vitamin B_12_ was identified. In addition, beef consumption was not found to be associated with overall dietary quality measured by the HEI-2015 score. After adjusting for multiple comparisons using FDR, fresh lean beef consumption was not associated with daily intake of total energy and sodium, whereas the other estimates remained statistically significant.

## 4. Discussion

Half of adults in the U.S. consume beef. Beef provides many nutrients that are essential to human health. However, different types of beef consumption in relation to nutrient intakes and diet quality is less documented. This study examined the influence of beef consumption on nutrient intakes and diet quality among U.S. adults using 12 years of data from a nationally representative nutrition survey. About 53.6%, 11.6%, 39.3%, and 6.7% of adults in the U.S. consumed beef, fresh beef, lean beef, and fresh lean beef, respectively (see Table 1). The prevalence of beef, fresh beef, lean beef, and fresh lean beef consumption differed by sex, age, race/ethnicity, education level, and obesity status (see Table 2). Beef, fresh beef, lean beef, and fresh lean beef consumption was associated with higher daily intakes of total energy, protein, sodium, choline, iron, selenium, zinc, phosphorus, and multiple B vitamins (See Table 3). Beef, fresh beef, and lean beef consumption, but not fresh lean beef consumption, was associated with higher saturated fat intake. Beef consumption was not associated with overall dietary quality measured by the HEI-2015 score.

This study confirmed the findings from previous work on the positive contribution of beef consumption on certain essential macronutrient and micronutrient intakes, such as protein, zinc, iron, and multiple B vitamins [5,6,7,8,9]. In addition, this study differentiated the influence between fresh and lean beef consumption. While overall beef consumption was most closely associated with intakes of those nutrients, higher nutrient intakes attributable to fresh beef, lean beef, and fresh lean beef consumption were fairly comparable. On the other hand, fresh beef, lean beef, and fresh lean beef consumption in particular, was associated with lower daily intakes of total energy, saturated fat, and sodium than beef consumption. For instance, an increase in beef consumption by one ounce-equivalent per day was associated with an increase in total energy by 46.1 kcal, saturated fat by 0.9 g, and sodium by 66.6 mg; whereas an increase in fresh lean beef consumption, by one ounce-equivalent per day, was associated with an increase in total energy by 23.3 kcal, saturated fat by 0.3 g (*p*-value > 0.05), and sodium by 39.1 mg (See Table 3). These findings suggest that beef consumers may increase the proportion of fresh and lean beef over total beef intake in order to capitalize on the nutritional gains from beef consumption, while minimizing the associated increase in energy, saturated fat, and sodium intake.

The prevalence of beef consumption was found to differ substantially in individual characteristics. Differential patterns in beef consumption, and more generally red meat consumption, have been extensively documented [24,25,26]. Dissimilarities in dietary traditions, and culture and biological differences, may partially explain the heterogeneous beef consumption patterns by sex, age, and race/ethnicity [24]. However, less is known about the role of education, marital status, and obesity in beef consumption. This study found that people with lower education level, married, and with obesity were more likely to consume beef products. A previous study documented that beef consumption is negatively associated with education level but positively associated with family size [27]. In contrast, poultry and fish consumption tended to increase with education level [24]. These findings may suggest that individuals with higher education could pay more attention to weight control and chronic disease prevention and thus choose to consume less red meat as a way to reduce caloric intake. Meat consumption was found to be associated with adiposity among U.S. adults [26]. It was possible that beef consumption increased obesity risk in the NHANES adult sample, but the current cross-sectional evaluation does not allow us to draw any causal conclusion due to potential confounding bias and reverse causality.

A few limitations of this study should be noted. NHANES is a probability sample of the U.S. non-institutionalized population, and the dietary intakes among patients in penal/mental facilities, institutionalized older adults, and/or military personnel on active duty are not represented. Dietary intake in NHANES was self-reported and subject to measurement error and social desirability bias [28]. First-difference estimator eliminated confounding bias from unobservable factors that remained constant, within-participant between the two dietary interviews but could not control for more transient factors such as daily variations in physical activity, appetite, or emotions. Compared to pooled cross-sectional estimators, the first-difference estimator is under-powered (less precision and larger standard error), because only a sub-sample consisting of individuals who alternated their beef consumption pattern between the 2 dietary recall days, contributed to the effect estimation, whereas those who did not consume any beef (or fresh, lean, and fresh lean beef) did not. Although the point estimates from regression analyses suggest some differences in the relationship between energy/nutrient intakes and beef consumption between alternative beef types, none of those differences were statistically significant. This study examined the short-term nutritional relevance of beef consumption; however, whether and to what extent changes in nutrient intakes and diet quality attributable to beef consumption, especially fresh and/or lean beef consumption, may influence long-term health and disease status, were beyond the scope of this study and warrant future research.

## 5. Conclusions

In conclusion, this study assessed the impact of beef consumption on energy/nutrient intakes and diet quality among U.S. adults, using data from a nationally representative nutrition survey. Beef, fresh beef, lean beef, and fresh lean beef consumption was associated with higher intakes of total energy, protein, sodium, choline, iron, selenium, zinc, phosphorus, and multiple B vitamins. Beef, fresh beef, and lean beef consumption were also associated with higher saturated fat intake. Beef consumers are advised to increase their portions of fresh and lean beef over total beef intake in an effort to maximize nutritional gains from beef consumption, while minimizing any resultant increase in energy, saturated fat, and sodium intake. This study has limitations pertaining to measurement error and observational study design. Future studies are warranted to examine the long-term health consequences of fresh and lean beef consumption.

## Figures and Tables

**Table 1:** Individual characteristics of 2005–2016 NHANES adult beef, fresh beef, lean beef, and fresh lean beef consumers and beef non-consumers.

Individual Characteristics	Beef Consumers Mean ± SD	Fresh Beef Consumers Mean ± SD	Lean Beef Consumers Mean ± SD	Fresh Lean Beef Consumers Mean ± SD	Beef Non-Consumers Mean ± SD
Sample Size	14,195	3023	10,530	1788	12,922
***Daily total energy/nutrient intake***					
Diet quality (HEI-2015)	49.82 ± 10.66	49.95 ± 10.48	49.96 ± 10.68	50.52 ± 10.76	53.26 ± 12.56 ***
Energy (kcal)	2209.89 ± 803.48	2181.35 ± 778.17	2209.14 ± 809.18	2140.70 ± 807.81	1977.73 ± 758.03 ***
Protein (g)	88.00 ± 34.00	86.48 ± 31.66	90.21 ± 34.24	86.80 ± 32.66	76.35 ± 32.77 ***
Saturated fat (g)	28.39 ± 13.50	27.96 ± 12.94	28.29 ± 13.59	26.72 ± 12.85	23.79 ± 12.50 ***
Sodium (mg)	3679.15 ± 1454.45	3592.11 ± 1358.5	3671.31 ± 1446.73	3559.64 ± 1411.27	3290.16 ± 1388.18 ***
Choline (mg)	353.92 ± 156.27	355.21 ± 145.17	364.43 ± 158.39	361.87 ± 154.85	305.71 ± 147.23 ***
Iron (mg)	16.01 ± 7.48	16.34 ± 8.17	16.09 ± 7.57	16.32 ± 8.88	14.52 ± 7.72 ***
Selenium (µg)	118.68 ± 50.46	115.57 ± 45.82	120.40 ± 50.74	115.48 ± 46.96	108.45 ± 50.71 ***
Zinc (mg)	13.27 ± 6.79	13.65 ± 6.78	13.79 ± 7.01	13.87 ± 7.54	10.02 ± 6.03 ***
Phosphorus (mg)	1424.80 ± 549.84	1399.32 ± 514.06	1435.37 ± 549.50	1394.92 ± 539.11	1316.67 ± 554.65 ***
Vitamin B_2_ (mg)	2.24 ± 1.06	2.26 ± 1.01	2.24 ± 1.07	2.24 ± 1.09	2.12 ± 1.06 ***
Vitamin B_3_ (mg)	26.81 ± 12.15	26.46 ± 11.97	27.14 ± 12.25	26.42 ± 13.08	24.76 ± 12.17 ***
Vitamin B_6_ (mg)	2.18 ± 1.20	2.13 ± 1.18	2.22 ± 1.21	2.18 ± 1.33	2.04 ± 1.22 ***
Vitamin B_12_ (µg)	5.75 ± 4.77	5.87 ± 4.75	5.95 ± 4.95	5.79 ± 4.72	4.73 ± 4.73 ***
***Daily energy/nutrient intake from beef products***					
Prevalence in sample (%)	53.56 ± 49.14	11.56 ± 31.50	39.27 ± 48.11	6.68 ± 24.60	46.44 ± 49.14 ***
Beef (oz)	2.11 ± 1.77	1.76 ± 1.34	2.17 ± 1.79	1.96 ± 1.42	/
Energy (kcal)	300.71 ± 252.61	217.88 ± 197.79	166.85 ± 129.17	159.56 ± 124.40	/
Protein (g)	22.88 ± 16.93	14.75 ± 11.49	20.59 ± 15.64	17.01 ± 12.43	/
Saturated fat (g)	5.68 ± 5.25	2.79 ± 2.50	3.93 ± 3.86	2.34 ± 2.12	/
Sodium (mg)	664.55 ± 625.76	310.85 ± 292.63	419.77 ± 444.96	341.50 ± 307.25	/
Choline (mg)	74.75 ± 58.72	52.96 ± 42.95	68.45 ± 53.74	61.64 ± 47.24	/
Iron (mg)	2.83 ± 2.25	1.77 ± 1.43	2.19 ± 1.81	1.97 ± 1.54	/
Selenium (µg)	25.71 ± 20.93	15.96 ± 14.66	22.11 ± 18.68	19.41 ± 16.79	/
Zinc (mg)	4.42 ± 3.33	2.98 ± 2.24	4.07 ± 3.12	3.25 ± 2.33	/
Phosphorus (mg)	232.36 ± 186.44	136.05 ± 107.28	185.62 ± 152.58	152.67 ± 112.49	/
Vitamin B_2_ (mg)	0.25 ± 0.24	0.15 ± 0.12	0.18 ± 0.17	0.16 ± 0.12	/
Vitamin B_3_ (mg)	5.30 ± 4.19	3.26 ± 2.74	4.60 ± 3.75	3.75 ± 3.04	/
Vitamin B_6_ (mg)	0.36 ± 0.29	0.20 ± 0.18	0.33 ± 0.27	0.25 ± 0.19	/
Vitamin B_12_ (µg)	1.74 ± 3.34	1.06 ± 0.80	1.48 ± 1.15	1.02 ± 0.75	/
***Sex (%)***					
Male	52.30 ± 47.89	52.72 ± 47.13	53.23 ± 47.87	51.18 ± 47.79	44.43 ± 49.28 ***
Female	47.70 ± 47.89	47.28 ± 47.13	46.77 ± 47.87	48.82 ± 47.79	55.57 ± 49.28 ***
***Age group (%)***					
18–64 years of age	82.76 ± 36.22	77.71 ± 39.29	82.27 ± 36.65	74.75 ± 41.53	81.22 ± 38.73
65 years of age and above	17.24 ± 36.22	22.29 ± 39.29	17.73 ± 36.65	25.25 ± 41.53	18.78 ± 38.73
***Race/ethnicity (%)***					
White, non-Hispanic	69.26 ± 44.24	72.41 ± 42.19	68.89 ± 44.42	70.21 ± 43.72	69.10 ± 45.83
African American, non-Hispanic	9.98 ± 28.73	10.75 ± 29.24	9.61 ± 28.28	10.33 ± 29.10	12.01 ± 32.23 **
Other race/multi-race, non-Hispanic	6.43 ± 23.51	5.68 ± 21.85	6.88 ± 24.29	6.24 ± 23.12	7.34 ± 25.86
Hispanic	14.34 ± 33.61	11.16 ± 29.72	14.62 ± 33.90	13.22 ± 32.38	11.56 ± 31.71 ***
***Education (%)***					
High school and below	40.99 ± 47.16	43.21 ± 46.76	41.06 ± 47.2	46.24 ± 47.66	35.51 ± 47.46 ***
College education and above	59.01 ± 47.16	56.79 ± 46.76	58.94 ± 47.2	53.76 ± 47.66	64.49 ± 47.46 ***
***Marital status (%)***					
Married	65.84 ± 45.47	65.58 ± 44.85	66.49 ± 45.29	65.81 ± 45.35	60.16 ± 48.55 ***
Divorced, separated, or widowed	16.98 ± 36.00	18.72 ± 36.82	16.84 ± 35.91	20.51 ± 38.60	20.12 ± 39.76 ***
Never married	17.18 ± 36.16	15.69 ± 34.33	16.67 ± 35.76	13.68 ± 32.85	19.72 ± 39.46 ***
***Income to poverty ratio (IPR) (%)***					
IPR < 130%	21.27 ± 39.24	20.53 ± 38.13	21.08 ± 39.13	22.15 ± 39.70	21.99 ± 41.07
130% ≤ IPR < 300%	29.67 ± 43.80	32.03 ± 44.04	29.38 ± 43.71	34.50 ± 45.44	27.64 ± 44.35
IPR ≥ 300%	49.06 ± 47.93	47.44 ± 47.13	49.54 ± 47.97	43.35 ± 47.37	50.38 ± 49.58
***Obesity (%)***					
Non-obese (BMI < 30)	62.99 ± 46.29	61.02 ± 46.04	62.91 ± 46.35	60.06 ± 46.82	66.19 ± 46.91 **
Obese (BMI ≥ 30)	37.01 ± 46.29	38.98 ± 46.04	37.09 ± 46.35	39.94 ± 46.82	33.81 ± 46.91 **
***Smoking (%)***					
Non-smoker	53.32 ± 47.83	50.94 ± 47.19	53.30 ± 47.87	48.67 ± 47.78	56.89 ± 49.11 ***
Former or current smoker	46.68 ± 47.83	49.06 ± 47.19	46.70 ± 47.87	51.33 ± 47.78	43.11 ± 49.11 ***
***Self-rated health (%)***					
Good or excellent health	83.05 ± 35.98	82.74 ± 35.67	83.11 ± 35.95	79.75 ± 38.42	82.97 ± 37.28
Fair or poor health	16.95 ± 35.98	17.26 ± 35.67	16.89 ± 35.95	20.25 ± 38.42	17.03 ± 37.28
***Chronic condition (%)***					
Diabetes	9.12 ± 27.60	9.85 ± 28.13	9.25 ± 27.79	10.71 ± 29.57	8.79 ± 28.08
Arthritis	25.69 ± 41.89	27.93 ± 42.35	25.30 ± 41.71	29.01 ± 43.38	25.27 ± 43.10
Coronary artery disease	3.54 ± 17.72	4.43 ± 19.43	3.58 ± 17.83	4.36 ± 19.53	3.13 ± 17.27
Stroke	2.68 ± 15.49	3.10 ± 16.35	2.73 ± 15.65	3.67 ± 17.97	2.89 ± 16.61
Cancer	9.87 ± 28.60	11.59 ± 30.22	10.39 ± 29.28	12.42 ± 31.53	10.50 ± 30.40

The NHANES multi-wave sampling design was accounted for in the estimates. HEI-2015 denotes Health Eating Index-2015 with possible score ranging from 0 (lowest daily diet quality) to 100 (highest daily diet quality). Two-sample t-tests for continuous variables and chi-squared tests for dichotomous variables were conducted between beef consumers and non-consumers, with statistical significance shown in the far right column; ** 0.001 ≤ *p* < 0.01; and *** *p* < 0.001./denotes not applicable.

**Table 2:** Adjusted odds ratios of beef, fresh beef, lean beef, and fresh lean beef consumption, 2005–2016 NHANES.

Individual Characteristics	Beef	Fresh Beef	Lean Beef	Fresh Lean Beef
***Sex***				
Male	Reference	Reference	Reference	Reference
Female	0.74 (0.69, 0.80) ***	0.83 (0.73, 0.95) **	0.75 (0.69, 0.81) ***	0.89 (0.74, 1.06)
***Age group***				
18–64 years of age	Reference	Reference	Reference	Reference
65 years of age and above	0.87 (0.76, 0.99) *	1.25 (1.07, 1.47) **	0.96 (0.85, 1.09)	1.46 (1.15, 1.85) **
***Race/ethnicity***				
White, non-Hispanic	Reference	Reference	Reference	Reference
African American, non-Hispanic	0.84 (0.75, 0.95) **	0.95 (0.81, 1.12)	0.85 (0.76, 0.96) **	0.94 (0.73, 1.20)
Other race/multi-race	0.94 (0.80, 1.11)	0.86 (0.67, 1.10)	1.14 (0.96, 1.35)	1.03 (0.76, 1.41)
Hispanic	1.21 (1.05, 1.39) **	0.80 (0.66, 0.96) *	1.24 (1.10, 1.41) **	0.98 (0.75, 1.26)
***Education***				
High school and below	Reference	Reference	Reference	Reference
College education and above	0.82 (0.75, 0.89) ***	0.83 (0.72, 0.95) **	0.87 (0.79, 0.95) **	0.81 (0.67, 0.99) *
***Marital status***				
Married	Reference	Reference	Reference	Reference
Divorced, separated, or widowed	0.81 (0.73, 0.90) ***	0.92 (0.77, 1.11)	0.83 (0.75, 0.92) **	0.95 (0.74, 1.22)
Never married	0.80 (0.71, 0.91) **	0.85 (0.71, 1.03)	0.81 (0.72, 0.90) ***	0.75 (0.61, 0.92) **
***Income to poverty ratio (IPR)***				
IPR < 130%	Reference	Reference	Reference	Reference
130% ≤ IPR < 300%	1.14 (1.01, 1.29) *	1.15 (0.95, 1.38)	1.08 (0.96, 1.20)	1.15 (0.92, 1.44)
IPR ≥ 300%	1.04 (0.91, 1.17)	0.99 (0.81, 1.20)	1.03 (0.92, 1.16)	0.89 (0.70, 1.12)
***Obesity***				
Non-obese (BMI < 30)	Reference	Reference	Reference	Reference
Obese (BMI ≥ 30)	1.18 (1.08, 1.28) ***	1.20 (1.03, 1.38) *	1.16 (1.07, 1.26) ***	1.22 (1.03, 1.43) *
***Smoking***				
Non-smoker	Reference	Reference	Reference	Reference
Former or current smoker	1.09 (1.01, 1.18) *	1.10 (0.95, 1.26)	1.06 (0.97, 1.16)	1.18 (1.00, 1.40)
***Self-rated health***				
Good or excellent health	Reference	Reference	Reference	Reference
Fair or poor health	0.90 (0.81, 1.00)	0.93 (0.80, 1.08)	0.91 (0.82, 1.01)	1.07 (0.89, 1.29)
***Chronic condition***				
Diabetes	1.01 (0.88, 1.16)	0.98 (0.76, 1.27)	1.04 (0.90, 1.20)	0.98 (0.73, 1.30)
Arthritis	1.09 (0.96, 1.23)	1.02 (0.87, 1.21)	1.02 (0.91, 1.14)	0.97 (0.80, 1.19)
Coronary artery disease	1.11 (0.88, 1.38)	1.13 (0.82, 1.55)	1.06 (0.85, 1.31)	0.96 (0.64, 1.42)
Stroke	0.94 (0.74, 1.19)	0.94 (0.69, 1.30)	0.97 (0.74, 1.27)	1.01 (0.65, 1.56)
Cancer	1.00 (0.89, 1.13)	1.07 (0.86, 1.33)	1.12 (0.99, 1.27)	1.11 (0.88, 1.41)

Logistic regressions were performed to estimate the adjusted odds ratios of beef consumption, accounting for the NHANES multi-wave complex sampling design. 95% confidence intervals are in parentheses. * 0.01 ≤ *p* < 0.05; ** 0.001 ≤ *p* < 0.01; and *** *p* < 0.001.

**Table 3:** Estimated effects of beef, fresh beef, lean beef, and fresh lean beef consumption on daily energy/nutrient intake and diet quality among U.S. adults, 2005–2016 NHANES.

Sample	Beef	Fresh Beef	Lean Beef	Fresh Lean Beef
Diet quality (HEI–2015)	–0.06 (–0.15, 0.02)	–0.00 (–0.20, 0.20)	0.05 (–0.04, 0.13)	0.19 (–0.04, 0.43)
Energy (kcal)	46.09 (39.20, 52.98) ***	39.57 (24.10, 55.04) ***	34.33 (27.07, 41.60) ***	23.32 (3.98, 42.66) *
Protein (g)	5.00 (4.70, 5.31) ***	4.09 (3.42, 4.77) ***	4.87 (4.56, 5.19) ***	4.22 (3.43, 5.02) ***
Saturated fat (g)	0.89 (0.76, 1.01) ***	0.75 (0.50, 1.00) ***	0.58 (0.44, 0.71) ***	0.28 (–0.00, 0.56)
Sodium (mg)	66.58 (52.37, 80.78) ***	63.43 (32.49, 94.37) ***	35.82 (21.90, 49.74) ***	39.12 (0.06, 78.19) *
Choline (mg)	18.90 (17.34, 20.45) ***	18.11 (14.73, 21.49) ***	19.35 (17.70, 21.01) ***	18.94 (14.66, 23.23) ***
Iron (mg)	0.57 (0.51, 0.64) ***	0.62 (0.43, 0.80) ***	0.45 (0.37, 0.52) ***	0.51 (0.27, 0.74) ***
Selenium (µg)	3.81 (3.30, 4.32) ***	3.42 (2.30, 4.54) ***	3.62 (3.10, 4.14) ***	3.79 (2.39, 5.20) ***
Zinc (mg)	1.39 (1.28, 1.49) ***	1.15 (0.93, 1.38) ***	1.32 (1.20, 1.44) ***	1.07 (0.76, 1.38) ***
Phosphorus (mg)	37.07 (32.57, 41.58) ***	37.53 (26.24, 48.82) ***	32.40 (27.47, 37.34) ***	34.38 (19.89, 48.86) ***
Vitamin B_2_ (mg)	0.03 (0.02, 0.04) ***	0.05 (0.03, 0.07) ***	0.02 (0.01, 0.03) ***	0.04 (0.01, 0.07) **
Vitamin B_3_ (mg)	0.92 (0.80, 1.04) ***	0.62 (0.32, 0.91) ***	0.88 (0.75, 1.01) ***	0.62 (0.23, 1.00) **
Vitamin B_6_ (mg)	0.10 (0.08, 0.11) ***	0.06 (0.04, 0.09) ***	0.10 (0.08, 0.11) ***	0.07 (0.03, 0.10) ***
Vitamin B_12_ (µg)	0.41 (0.32, 0.49) ***	0.28 (0.13, 0.44) ***	0.38 (0.29, 0.47) ***	0.16 (−0.05, 0.38)

Individual-level data from the NHANES 2005–2016 waves. First-difference estimators were used to estimate the effects of beef consumption on daily dietary intake and diet quality among U.S. adults, adjusting for whether the consumption was on a weekday or weekend and accounting for the NHANES multiyear complex survey design. HEI-2015 denotes Healthy Eating Index-2015 with possible score ranging from 0 (lowest daily diet quality) to 100 (highest daily diet quality). 95% confidence intervals are in parentheses. * 0.01 ≤ *p* < 0.05; ** 0.001 ≤ *p* < 0.01; and *** *p* < 0.001.

## References

[1] Industry Statistics. http://www.beefusa.org/beefindustrystatistics.aspx.

[2] Young V.R. (1990). Amino acids and protein in relation to the nutrition of elderly people. Age Ageing.

[3] Micha R., Wallace S., Mozaffarian D. (2010). Red and processed meat consumption and risk of incident coronary heart disease, stroke, and diabetes: A systematic review and meta-analysis. Circulation.

[4] U.S. Department of Health and Human Services and U.S. Department of Agriculture (2015). 2015–2020 Dietary Guidelines for Americans. 8th Edition. http://health.gov/dietaryguidelines/2015/guidelines.

[5] Nicklas T.A., O’Neil C.E., Zanovec M., Keast D.R., Fulgoni V.L. (2012). Contribution of beef consumption to nutrient intake, diet quality, and food patterns in the diets of the US population. Meat Sci..

[6] Bowen J., Baird D., Syrette J., Noakes M., Baghurst K. (2012). Consumption of beef/veal/lamb in Australian children: Intake, nutrient contribution and comparison with other meat, poultry and fish categories. Nutr. Diet..

[7] O’Neil C.E., Zanovec M., Keast D.R., Fulgoni V.L., Nicklas T.A. (2011). Nutrient contribution of total and lean beef in diets of US children and adolescents: National Health and Nutrition Examination Survey 1999–2004. Meat Sci..

[8] Zanovec M., O’Neil C., Keast D., Fulgoni V., Nicklas T. (2010). Lean beef contributes significant amounts of key nutrients to the diets of US adults: National Health and Nutrition Examination Survey 1999–2004. Nutr. Res..

[9] Zanovec M., O’Neil C.E., Keast D.R., Fulgoni V.L., Nicklas T.A. (2010). Improved nutrient intake and diet quality associated with lean beef consumption in the US: National Health and Nutrition Examination Survey (NHANES) 1999–2004. FASEB J..

[10] Sharma S., Sheehy T., Kolonel L.N. (2013). Contribution of meat to vitamin B12, iron and zinc intakes in five ethnic groups in the USA: Implications for developing food-based dietary guidelines. J. Hum. Nutr. Diet..

[11] Kim K., Hyeon J., Lee S.A., Kwon S.O., Lee H., Keum N., Lee J.K., Park S.M. (2017). Role of total, red, processed, and white meat consumption in stroke incidence and mortality: A systematic review and meta-analysis of prospective cohort studies. J. Am. Heart Assoc..

[12] Tian S., Xu Q., Jiang R., Han T., Sun C., Na L. (2017). Dietary protein consumption and the risk of type 2 diabetes: A systematic review and meta-analysis of cohort studies. Nutrients.

[13] Gonzalez-Garcia S., Esteve-Llorens X., Teresa Moreira M., Feijoo G. (2018). Carbon footprint and nutritional quality of different human dietary choices. Sci. TOTAL Environ..

[14] Van Mierlo K., Rohmer S., Gerdessen J.C. (2017). A model for composing meat replacers: Reducing the environmental impact of our food consumption pattern while retaining its nutritional value. J. Clean. Prod..

[15] McNeill S.H., Van Elswyk M.E. (2012). Red meat in global nutrition. Meat Sci..

[16] Binnie M.A., Barlow K., Johnson V., Harrison C. (2014). Red meats: Time for a paradigm shift in dietary advice. Meat Sci..

[17] Chowhury R., Warnakula S., Kunutsor S., Crowe F., Ward H., Johnson L., Angelantonio E. (2014). Association of dietary, circulating, and supplement fatty acids with coronary risk: A systematic review and meta-analysis. Ann. Intern. Med..

[18] McNeill S.H. (2014). Inclusion of red meat in healthful dietary patterns. Meat Sci..

[19] Cotton P., Subar A., Friday J., Cook A. (2004). Dietary sources of nutrients among US adults, 1994 to 1996. J. Am. Diet Assoc..

[20] NHANES–National Health and Nutrition Examination Survey Homepage. https://www.cdc.gov/nchs/nhanes/index.htm.

[21] Overview & Background of The Healthy Eating Index. https://epi.grants.cancer.gov/hei/.

[22] Krebs-Smith S.M., Pannucci T.E., Subar A.F., Kirkpatrick S.I., Lerman J.L., Tooze J.A., Wilson M.M., Reedy J. (2018). Update of the Healthy Eating Index: HEI-2015. J. Acad. Nutr. Diet..

[23] Obesity and Overweight. https://www.who.int/news-room/fact-sheets/detail/obesity-and-overweight.

[24] Daniel C., Cross A.J., Koebnick C., Sinha R. (2010). Trends in meat consumption in the USA. Public Health Nutr..

[25] Guenther P.M., Jensen H.H., Batres-Marquez S.P., Chen C.-F. (2005). Sociodemographic, knowledge, and attitudinal factors related to meat consumption in the united states. J. Am. Diet. Assoc..

[26] Wang Y., Beydoun M.A., Caballero B., Gary T.L., Lawrence R. (2010). Trends and correlates in meat consumption patterns in the US adult population. Public Health Nutr..

[27] Ward R.W. (2009). The Beef Checkoff Programs and Their Impact on U.S. Beef Demand.

[28] Hebert J.R., Hurley T.G., Peterson K.E., Resnicow K., Thompson F.E., Yaroch A.L. (2008). Social Desirability trait influences on self-reported dietary measures among diverse participants in a multicenter risk factor trial. J. Nutr..

